# Epigenetic, Genetic and Environmental Interactions in Esophageal Squamous Cell Carcinoma from Northeast India

**DOI:** 10.1371/journal.pone.0060996

**Published:** 2013-04-15

**Authors:** Fazlur Rahman Talukdar, Sankar Kumar Ghosh, Ruhina Shirin Laskar, Rosy Mondal

**Affiliations:** Department of Biotechnology, Assam University, Silchar, Assam, India; Chinese Academy of Medical Sciences, China

## Abstract

**Background:**

Esophageal squamous cell carcinoma (ESCC) develops as a result of complex epigenetic, genetic and environmental interactions. Epigenetic changes like, promoter hypermethylation of multiple tumour suppressor genes are frequent events in cancer, and certain habit-related carcinogens are thought to be capable of inducing aberrant methylation. However, the effects of environmental carcinogens depend upon the level of metabolism by carcinogen metabolizing enzymes. As such key interactions between habits related factors and carcinogen metabolizing gene polymorphisms towards modulating promoter methylation of genes are likely. However, this remains largely unexplored in ESCC. Here, we studied the interaction of various habits related factors and polymorphism of *GSTM1*/*GSTT1* genes towards inducing promoter hypermethylation of multiple tumour suppressor genes.

**Methodology/Principal Findings:**

The study included 112 ESCC cases and 130 age and gender matched controls. Conditional logistic regression was used to calculate odds ratios (OR) and multifactor dimensionality reduction (MDR) was used to explore high order interactions. Tobacco chewing and smoking were the major individual risk factors of ESCC after adjusting for all potential confounding factors. With regards to methylation status, significantly higher methylation frequencies were observed in tobacco chewers than non chewers for all the four genes under study (p<0.01). In logistic regression analysis, betel quid chewing, alcohol consumption and null *GSTT1* genotypes imparted maximum risk for ESCC without promoter hypermethylation. Whereas, tobacco chewing, smoking and *GSTT1* null variants were the most important risk factors for ESCC with promoter hypermethylation. MDR analysis revealed two predictor models for ESCC with promoter hypermethylation (Tobacco chewing/Smoking/Betel quid chewing/*GSTT1* null) and ESCC without promoter hypermethylation (Betel quid chewing/Alcohol/*GSTT1*) with TBA of 0.69 and 0.75 respectively and CVC of 10/10 in both models.

**Conclusion:**

Our study identified a possible interaction between tobacco consumption and carcinogen metabolizing gene polymorphisms towards modulating promoter methylation of tumour suppressor genes in ESCC.

## Introduction

The Esophageal cancer (EC) is the sixth most common cancer in men worldwide with distinct geographical differences in its incidence rate and pattern. The incidence and mortality rate of EC is highest in certain Asian countries, stretching from Northern Iran through the central Asian republics to North-Central China, referred to as the “esophageal cancer belt.” Around 90% of the esophageal cancers in these areas are squamous cell carcinomas (SCCs) and are thought to develop as a result of complex interactions between environmental, genetic and epigenetic factors [1]. However, these interactions are not well understood in ESCC. Environmental and dietary factors like smoking and smokeless tobacco consumption, betel quid chewing, alcohol intake, poor nutrition, etc., are considered to be associated with ESCC in the high risk areas [2], [3]. Moreover, polymorphism in various carcinogen metabolizing genes modulates the effect of these environmental carcinogens and further increases the risk of ESCC [4]. The interaction of tobacco related carcinogens and carcinogen metabolizing genes like *GSTM1, GSTT1,* etc., were found to modify the effect of tobacco exposure thereby increasing the susceptibility for developing ESCC [5], [6].

Epigenetic events like aberrant DNA methylation of tumours suppressor genes (TSGs) are considered as important factors in development and progression of ESCC. The TSGs involved in different cellular pathways like cell cycle regulation (*p16*), apoptosis (*DAPK*), DNA repair (*BRCA1*) and protection of DNA (*GSTP1)* [7], [8]. Increasing evidence are growing that tobacco smoke associated carcinogens and carcinogen metabolizing gene polymorphisms are capable of modulating DNA methylation in cultures, animal models as well as certain tobacco-related cancers like lung cancer[9]–[12]. Cigarette smoke has also been found to induce promoter methylation of particular genes in esophageal epithelial and ESCC cell lines; however, no study involving human subjects were carried out [13], [14]. Furthermore, null genotype of *GSTM1* gene was associated with an increased susceptibility of CpG island hypermethylation in gastric-mucosa [15]. Although, ESCC is one of the most important tobacco related cancers, but the interaction of smoking and smokeless tobacco, carcinogen metabolizing gene polymorphisms and aberrant DNA methylation in ESCC has remained largely unexplored.

This study is conducted on a unique population of Northeast (NE) India, where tobacco related habits like tobacco chewing; beedi and cigarette smoking are common. Moreover, consumption of a combination of areca nut, betel leaf, slaked lime with or without tobacco, called ‘betel quid (BQ)’ or locally as ‘pan’ or ‘tambul’ is customary in this concerned population. The Assam and Mizoram states of NE-India are among the highest incidence region of esophageal cancer, with an age-adjusted rate of around 17/100000 to 27 per/100000 population [16]. Although, previous studies on the risk factors of ESCC in NE-Indian population specify the association of tobacco and BQ chewing with its carcinogenesis, but very little is established about the environmental, genetic or epigenetic risk factors [17]. Moreover, no studies were conducted on DNA methylation signatures of the ESCC patients in this population. Here, we analyzed the association of various habits related factors (like tobacco chewing, beedi and cigarette smoking, BQ chewing and alcohol consumption) and carcinogen metabolizing gene polymorphisms (*GSTM1*, *GSTT1*) in ESCC and also stratified by promoter hypermethylation of TSGs, like *p16, DAPK, BRCA1* and *GSTP1* by logistic regression analysis. Multifactor dimensionality reduction (MDR) and false-positive report frequencies (FPRP) were used to predict high order interactions involving those factors of Epigenetic, Genetic and Environmental in ESCC from NE Indian population.

## Materials and Methods

### Study Population

Surgically excised cancer tissues (prior to chemo-radiation therapy), biopsy specimen or formalin fixed paraffin-embedded tissues of 112 histopathologically confirmed ESCC patients from different cancer hospitals of NE India during January 2011 to October 2012 were included. Histological proven normal margins of 30 patients undergoing curative surgery for ESCC were considered for comparison. Oral swabs from inner cavity of 130 age and gender matched healthy controls were also collected. Both cases and controls with family history of esophageal or other cancers were excluded. All possible precautions were taken to avoid any cross-contamination while collecting as well as processing of the samples.

### Ethics Statement

The study was approved by the Institutional Review Board of Cachar Cancer Hospital and Research Centre (http://cacharcancerhospital.org), Assam, and the written consents were taken from the subjects (IRB No: IRB/CCHRC/01/2010).

### Exposure to Environmental Factors

Demographic and habit related data such as dietary factors, life time betel quid and tobacco chewing, smoking and alcohol consumption details, family history of cancer in first degree relatives, co-morbid conditions and clinical features of esophageal cancer with complete medical history were collected using a structured questionnaire. Tobacco and betel quid chewing, smoking and alcohol consumption were included in the analysis as ever or never. Betel quid chewing is defined as betel leaf, areca nut (raw/dried/fermented), slaked lime without tobacco. Similarly, tobacco chewing is the chewing of dried tobacco leaf, zarda (moist or dry tobacco mixed with variety of colourings and spices) and khaini (tobacco mixed with lime and flavours) either alone or with betel quid. For tobacco and betel quid chewing, subjects who did not chew or chewed less than 100 times or were non-chewers during the collection of information were considered as never chewers. Subjects who do not smoke or smoke less than 100 cigarettes/beedis in their lifetime or currently non-smokers were considered as never smokers. Majority of the subjects belonged to rural background with agriculture, business or small jobs, which does not radically expose them to occupational hazards.

### DNA Extraction

Genomic DNA was isolated from cancerous biopsy samples, surgically excised cancer tissues and inner oral swabs by standard phenol/chloroform protocol [18]. The isolated DNA was then dissolved in Tris-EDTA buffer and stored at −80°C for further analysis. Genomic DNA from formalin fixed paraffin embedded tissues were isolated using Bioline Isolate Genomic DNA minikit (Bioline, UK) following manufacturer’s instructions.

### Genotyping of *GSTT1* and *GSTM1*

*GSTM1* -*GSTT1* gene polymorphism using *CYP1A1* gene as internal control using forward (F) and reverse (R) primers for the amplification *GSTT1* F5′-TTCCTTACTGGTCCTCACATTCTC-3′ and R 5′-TCACGGGATCATGGCCAGCA-3′, *GSTM1* F5′-GAACTCCCTGAAAAGCTAAAGC-3′ and R5′-GTTGGGCTCAAATATACGGTGG-3′, *CYP1A1* F5′- GAACTGCCACTTCAGCTGTCT and R5′-GCTGCATTTGGAAGTGCTC respectively [19].

### Bisulfite Conversion of DNA and Methyl Specific PCR

Bisulfite modification of genomic DNA was done by using Imprint® DNA Modification kit (Sigma-Aldrich), following manufacturer’s instructions. Promoter methylation status of *p16*, *DAPK, GSTP1* and *BRCA1* was determined by Methylation Specific PCR (MSP) following the primers and conditions [20]. We used two sets of primer, one specific for methylated DNA at the promoter region of each gene and the other set specific for unmethylated DNA. DNA from peripheral blood lymphocytes treated with SssI methyltranferase was used as positive control and DNA from peripheral blood lymphocytes of healthy individuals were used as negative control for methylated genes and viewed in 3% agarose gel.

### Statistical Analysis

Association between the environmental, genetic and epigenetic factors were carried out by conditional logistic regression and p-value <0.05 was considered statistically significant. Comparison between categorical data was done by Fisher’s exact test or Chi-square tests as appropriate.

### Multifactor Dimensionality Reduction (MDR) Analysis

The MDR software package (www.multifactordimensionalityreduction.org) was used to detect the gene-gene and gene-environment interactions. MDR is a model-free, non-parametric approach that can detect higher order interactions even in a small population by reducing the dimensionality of multi-locus information to identify the polymorphisms or factors associated with an increased risk of disease. This helps in overcoming the limitations of low statistical power due to very high degrees of freedom when using logistic regression in studying higher order interactions. The best model for each order of interaction was selected by maximum cross validation consistency (CVC) and testing balanced accuracy (TBA). Interaction models showing highest TBA and CVC was further tested by 1000 folds permutation tests and χ^2^ test at 0.05% significance levels.

### Interaction Entropy Graphs

The entropy-based analysis included in the MDR software package was used to determine synergistic and non-synergistic interactions among the variables. The graphs comprise of nodes containing entropy removed by individual variables and connections joining them pairwise showing entropy of interaction between them. Positive entropy signifies synergy and negative entropy indicate redundancy, whereas, zero entropy indicates independence.

### False Positive Report Probability (FPRP)

Results of higher order gene-environment interactions are often affected by the risk of being false positives. In order to detect the false positive report probability (FPRP) and the consistency of our MDR results, we used odds ratios and 95% confidence intervals from MDR analysis, observed p-values and power to detect odds ratios (ORs) of 1.5 and 2.0 in a Bayesian approach [21]. Considering a small sample size as ours, the FPRP was computed using prior probabilities ranging from 0.25 to 10^−5^ with a preset FPRP for noteworthiness equal to 0.5.

## Results

### Characteristics of the Subjects Under Study

The study comprised of 63% males and 37% females in cases and 66% males and 34% females in controls. The median age was 55 years (range = 30–76 years) and 57 years (range = 25–85 years) for cases and controls respectively. Most of the subjects belonged to rural areas (73% cases and 79% controls) and had weak financial conditions. Betel quid chewing with or without tobacco was the most prevalent habit as it is customary in the concerned population of NE India. Among the subjects, 39.28% and 41.07% of the cases and 31.53% and 29.23% of the controls had null variants of *GSTM1* and *GSTT1* genes respectively.

### Promoter Methylation Profile

**Figure 1:**
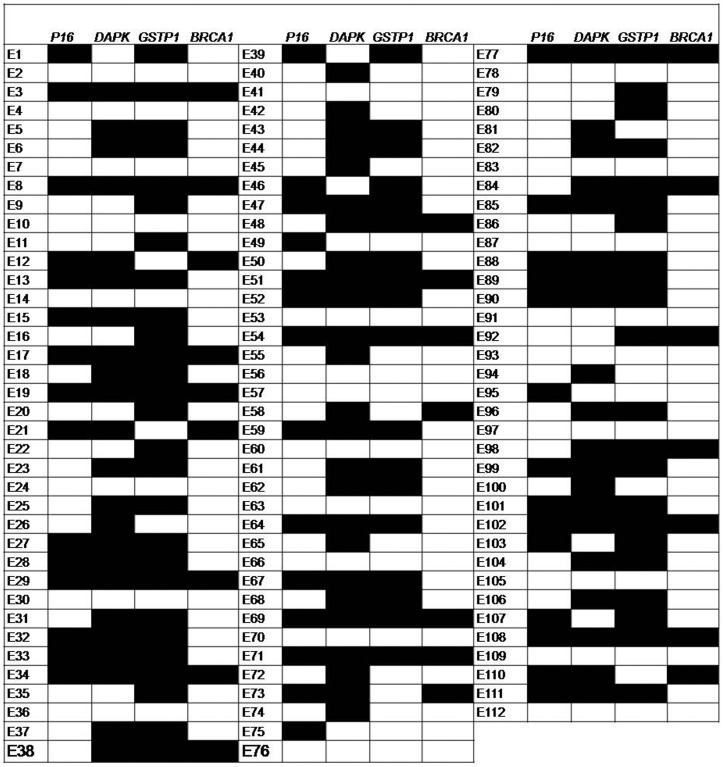
Promoter methylation profile of *p16, DAPK, GSTP1* and *BRCA1* genes of 112 ESCC patients. Each column and row represent the respective gene indicated on top and individual patients. The number indicated on the left corresponds to the patient number. Black rectangles are methylated samples, and white rectangles are unmethylated samples.

Promoter methylation status corresponding to the *p16, DAPK, GSTP1* and *BRCA1* genes of the 112 ESCC samples is shown in Figure 1. The frequency of promoter methylation was 37.5% (42/112), 61.60% (69/112), 58.92% (66/112) and 20.53% (23/112) for *p16*, *DAPK, GSTP1* and *BRCA1* genes respectively. However, methylation analysis of non-malignant tissues of 30 patients undergoing curative surgery has shown a much lower frequency of methylation (6.66% for *p16* and 13.33% for *DAPK*, 16.66% for *GSTP1* and 0% for *BRCA1*) as compared to their corresponding tumour tissues (46.7%, 63.3%, 63.3% and 20% for *p16, DAPK*, *GSTP1* and *BRCA1* respectively) shown in Figure S1.

**Figure 2:**
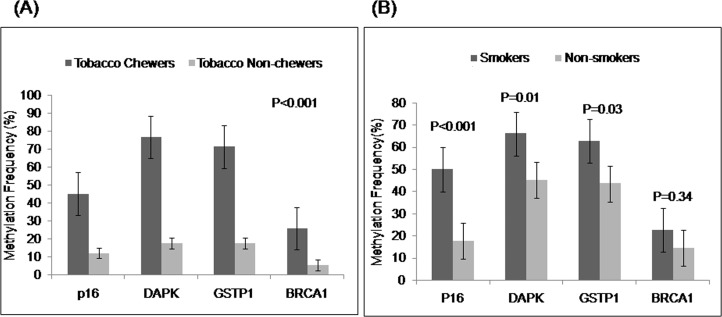
Methylation frequencies of patients stratified by tobacco chewing and smoking. (A) The paired bars depicts the comparison of methylation frequencies for individual tumour suppressor genes in tobacco chewers and non-chewers, along with standard error bars. (B) Comparison of methylation frequencies for individual tumour suppressor genes in smokers and non-smokers, along with standard error bars.

The methylation index (MI) (calculated as the ratio of the number of methylated promoters and total number of promoters under study) ranged from 0 to 1.27 of the 112 (24.10%) patients had MI of 0.46 (41.07%) had MI of 0.25–0.5 and 39 (34.82%) had MI of 0.75–1.0. The frequency of promoter methylation was significantly higher in tobacco chewers as compared to non-chewers for all the genes under study (p<0.001, Figure 2A) whereas, smokers had higher frequency of *p16, DAPK* and *GSTP1* methylation than non-smokers (Figure 2B).

### Logistic Regression Analysis of Risk Factors for ESCC

Table 1 summarizes the distribution and individual effects of the various environmental and genetic factors under study. Tobacco chewing and smoking (adjusted OR = 2.63 [95% CI = 1.53–4.5] for tobacco chewing and adjusted OR = 2.50 [95% CI = 1.46–4.16] for smoking) were found to be the major risk factors for ESCC after adjusting for the potential confounding factors like age, gender, betel quid chewing and alcohol consumption. Although, the practice of betel quid chewing was very common, but it was not found to be significantly associated with ESCC independently. Similarly, alcohol consumption had shown only modest association with ESCC (adjusted OR = 1.23[95% CI = [0.67–2.46]). Null variants of *GSTM1* and *GSTT1* had a moderately increased risk of ESCC; however, the risk was significantly higher for *GSTT1* null variants only (Table 1).

**Table 1:** Odds Ratio of the major risk factors in esophageal squamous cell carcinoma (ESCC).

Factors	Ca/Co	Crude OR [95% CI]	P-value	Adjusted OR*[95% CI]	P-value
**Betel Quid**					
Chewers	74/82	1.13 [0.67–1.93]	0.62	0.95 [0.54–1.65]	0.86
Non-chewers	38/48	1 (ref)		1 (ref)	
**Tobacco**					
Chewers	73/56	2.47 [1.46–4.16]	0.0007	2.63 [1.53–4.5]	0.0004
Non-chewers	39/74	1 (ref)		1 (ref)	
**Smoking**					
Smokers	62/46	2.26 [1.34–3.80]	0.002	2.50 [1.46–4.28]	0.0008
Non-smokers	50/84	1 (ref)		1 (ref)	
**Alcohol**					
Drinkers	26/25	1.26 [0.68–2.35]	0.44	1.23 [0.67–2.46]	0.43
Non-drinkers	86/105	1 (ref)		1 (ref)	
***GSTM1***					
−	44/40	1.45 [0.85–2.47]	0.16	1.44 [0.84–2.47]	0.17
+	68/90	1 (ref)		1 (ref)	
***GSTT1***					
−	46/37	1.75 [1.02–2.99]	0.04	1.74 [1.01–2.98]	0.04
+	66/93	1 (ref)		1 (ref)	

OR = Odds Ratio, CI = confidence Interval, (ref) = reference group, Ca = Cases, Co = controls.

* Adjusted for age, gender, betel quid chewing, tobacco chewing, smoking and alcohol consumption.

### Risk Assessment of ESCC with Promoter Hypermethylation

The effects of environmental and genetic polymorphisms on ESCC stratified by promoter hypermethylation status as compared to controls are shown in table 2. Both smokeless and smoked forms of tobacco consumption had the highest risk of ESCC with promoter methylation of all the four genes under study. Tobacco chewing had 4.84, 5.69, 5.28 and 6.27 folds increased risk, and smoking had 5.14, 2.67, 2.63 and 2.84 folds risk of ESCC with promoter hypermethylation of *p16, DAPK, GSTP1* and *BRCA1* genes respectively. In addition, significant association was observed between *GSTM1* null genotypes and promoter methylation of *p16, DAPK* and *GSTP1* genes. Null genotypes of *GSTT1* gene had an association with *p16* and *BRCA1* methylation only.

**Table 2:** Association of habit related factors and polymorphisms of *GSTM1*, *GSTT1* genes in ESCC with and without promoter hypermethylation of *p16*, *DAPK, GSTP1 and BRCA1* genes.

Habits and Gene polymorphism	Cases with TSG promoter methylationVs. Controls				Cases without TSG promoter methylationVs. Controls			
	*p16*	*DAPK*	*GSTP1*	*BRCA1*	*p16*	*DAPK*	*GSTP1*	*BRCA1*
	OR[95% CI]	OR[95% CI]	OR[95% CI]	OR[95% CI]	OR[95% CI]	OR[95% CI]	OR[95% CI]	OR[95% CI]
**Betel quid**								
Chewers	0.46[0.23–0.93]	0.80[0.44–1.46]	0.79[0.43–1.45]	0.20[0.07–0.55]*	2.34[1.17–4.6]*	2.56[1.09–5.97]*	2.10[0.96–4.62]	1.89[1.03–3.47]*
Non-chewers	1(ref)	1(ref)	1(ref)	1 (ref)	1(ref)	1(ref)	1(ref)	1(ref)
**Tobacco**								
Chewers	4.84[2.14–10.94]**	5.69[2.83–11.41]**	5.28[2.62–10.64]**	6.27[2.02–19.48]**	1.66[0.92–2.98]	0.86[0.42–1.74]	1.01[0.51–2.00]	2.03[1.17–3.53]*
Non-chewers	1(ref)	1(ref)	1(ref)	1 (ref)	1(ref)	1(ref)	1(ref)	1(ref)
**Smoking**								
Smokers	5.14[2.36–11.18]**	2.67[1.46–4.87]**	2.63[1.43–4.84]**	2.84[1.14–7.06]*	1.45[0.80–2.62]	1.74[0.86–3.50]	1.82[0.92–3.60]	2.13[1.23–3.7]**
Non-smokers	1(ref)	1(ref)	1(ref)	1 (ref)	1(ref)	1(ref)	1(ref)	1(ref)
**Alcohol**								
Drinker	0.84[0.3–2.11]	0.88[0.41–1.89]	0.75[0.33–1.67]	0.88[0.27–2.82]	1.80[0.91–3.52]	2.02[0.93–4.39]	2.24[1.06–4.72]*	1.37[0.72–2.64]
Non-drinker	1(ref)	1(ref)	1(ref)	1 (ref)	1(ref)	1(ref)	1(ref)	1(ref)
***GSTM1***								
***−***	2.47[1.21–5.03]*	2.45[1.34–4.47]**	2.25[1.22–4.13]**	1.73[0.70–4.27]	1.03[0.55–1.93]	0.77[0.35–1.68]	0.62[0.28–1.38]	1.39[0.78–2.45
***+***	1(ref)	1(ref)	1(ref)	1 (ref)	1(ref)	1(ref)	1(ref)	1(ref)
***GSTT1***								
−	2.76[1.35–5.65]**	1.61[0.87–2.99]	1.53[0.81–2.86]	3.26[1.31–8.10]*	1.82[0.94–3.49]	1.80[0.88–3.70]	2.11[1.05–4.22]*	1.48[0.83–2.63]
+	1(ref)	1(ref)	1(ref)	1(ref)	1(ref)	1(ref)	1(ref)	1(ref)

TSGs = Tumour Suppressor Genes, OR = Odds Ratio, CI = confidence Interval,*p</ = 0.05, **p<0.01, (ref) = reference group. Adjusted for age, gender, betel quid chewing, tobacco chewing, smoking and alcohol consumption.

Further classifying the cases according to methylation index (MI), betel quid chewing was the strongest individual risk factor for ESCC with zero methylation index (OR = 4.68 [95% CI = 1.33–16.37]), followed by null *GSTT1* genotype and alcohol consumption (OR = 2.40 [95% CI = 1.16–6.30] and 2.70 [95% CI = 1.00–6.04] respectively) compared to controls (Table 3). Betel quid and tobacco chewing had the highest risk of ESCC having methylation index 0.25–0.50, with an odds ratio of 2.34 and 3.63 respectively. However, cases with higher methylation index (0.75–1.0) had strongest associations with tobacco consumption, both chewing (OR = 6.04 [95% CI = 2.4–14.68]) and smoking (OR = 5.29 [95%CI = 2.37–11.82]). Moreover, null variants of *GSTM1* and *GSTT1* had an elevated risk of ESCC with methylation index of 0.75–1.0 than controls (Table 3).

**Table 3:** Interaction of betel quid and tobacco chewing, smoking, polymorphism of *GSTM1*, *GSTT1* genes with promoter methylation index of 0, 0.25–0.50 and 0.75–1.00 in ESCC.

	Cases with Methylation index Vs. controls	P-value	Cases with Methylation index0.25–0.5 Vs. controls	P-value	Cases with Methylation index0.75–1.0 Vs. controls	P-value
	OR[95% CI]		OR[95% CI]		OR[95% CI]	
**Betel quid**						
Chewers	4.68[1.33–16.37]	0.004	2.34[1.03–5.27]	0.04	0.29[0.13–0.62]	0.001
Non-chewers	1(ref)		1(ref)		1(ref)	
**Tobacco**						
Chewers	0.462 [0.18–1.17]	0.10	3.63[1.72–7.66]	0.0007	6.04[2.4–14.68]	0.0001
Non-chewers	1(ref)		1(ref)		1(ref)	
**Smoking**						
Smokers	1.46[0.63–3.38]	0.37	1.46 [0.73–2.91]	0.28	5.29[2.37–11.82]	<0.0001
Non-smokers	1(ref)		1(ref)		1(ref)	
**Alcohol**						
Drinkers	2.47[1.00–6.04]	0.04	1.35 [0.60–3.04]	0.45	0.61[0.21–1.73]	0.36
Non-drinkers	1(ref)		1(ref)		1(ref)	
***GSTM1***						
-	0.51[0.18–1.44]	0.20	1.24[0.60–2.53]	0.55	2.91[1.39–6.06]	0.004
+	1(ref)		1(ref)		1(ref)	
***GSTT1***						
-	2.70[1.16–6.30]	0.02	0.54[0.21–1.27]	0.16	4.02[1.90–8.50]	0.003
+	1(ref)		1(ref)		1(ref)	

TSGs = Tumour Suppressor Genes, OR = Odds Ratio, CI = Confidence Interval, (ref) = reference group.

Adjusted for age, gender, betel quid chewing, tobacco chewing, smoking and alcohol consumption.

### MDR Analysis

The best predictive models of interaction between environmental and genetic parameters up to four orders of interaction, showing the CVC, training and testing balanced accuracy and p-value of chi-square and 1000 fold permutation test are summarized in Table 4. The analysis included the whole data set of 112 ESCC patients and 130 controls, patients stratified by promoter methylation status and methylation index separately. For ESCC, tobacco chewing was the foremost 1^st^ order model with TBA = 0.67, TrBA = 0.67 and CVC = 10. The best model for 2^nd^ order interaction was tobacco chewing and alcohol consumption (OR = 5.01 [95% CI = 2.54–9.88], TBA = 0.62, TrBA = 0.67 and CVC = 7), while the best 3^rd^ order interaction model included tobacco chewing, smoking and null *GSTT1* genotype (OR = 8.98 [95% CI = 4.15–19.43]). The 4^th^ order interaction model of tobacco, betel quid chewing, smoking and *GSTT1* null genotype was the best model with an odds ratio of 8.66 [95% CI = 4.41–17.01, p<0.0001]; maximum TBA (0.70), TrBA (0.73) and CVC (10/10). For ESCC with promoter hypermethylation, tobacco chewing imparted the highest risk individually (OR = 4.59 [95% CI = 2.47–8.50]) with maximum TBA (0.67), TrBA (0.67) and CVC (10/10). The best model with highest TBA and CVC consisted of tobacco and betel quid chewing, smoking and *GSTT1* null interaction (OR = 8.49 [95% CI = 4.23–17.05], TBA = 0.69, TrBA = 0.73 and CVC = 10). For ESCC without promoter hypermethylation, betel quid chewing was the most significant individual risk factor identified in MDR analysis (OR = 4.68 [95% CI = 1.33–16.37], TBA = 0.60, TrBA = 0.63 and CVC = 10). Betel quid chewing, alcohol consumption and null *GSTT1* interaction was the best model predicted with a maximum TBA (0.75), TrBA (0.75), CVC (10/10) and an odds ratio of 9.88 [95% CI = 3.67–26.54].

**Table 4:** MDR Analysis.

Data set	Order of interaction	Model	P-value (χ^2^-test)	Training Balance Accuracy	p-value*	Testing Balance Accuracy	p-value*	CVC
ESCC	1^st^ order	Tob	<0.0001	0.67	<0.0001	0.67	0.11	10
	2^nd^ order	Tob/Alc	<0.0001	0.67	<0.0001	0.62	0.24	7
	3^rd^ order	Tob/Smk/*GSTT1*	<0.0001	0.70	<0.0001	0.63	0.22	8
	**4^th^ order**	**Bq/Tob/Smk/** ***GSTT1***	**<0.0001**	**0.73**	**<0.0001**	**0.70**	**0.08**	**10**
ESCC with promoter hypermethylation	1^st^ order	Tob	<0.0001	0.67	<0.0001	0.67	0.11	10
	2^nd^ order	Tob/*GSTT1*	<0.0001	0.67	<0.0001	0.63	0.22	9
	3^rd^ order	Tob/Smk/*GSTT1*	<0.0001	0.70	<0.0001	0.63	0.21	6
	**4^th^ order**	**Tob/Bq/Smk/** ***GSTT1***	**<0.0001**	**0.73**	**<0.0001**	**0.69**	**0.08**	**10**
ESCC without promoter hypermethylation	1^st^ order	Bq	0.009	0.63	0.01	0.60	0.50	9
	2^nd^ order	Bq/Tob	0.0003	0.68	0.0006	0.63	0.42	6
	**3^rd^ order**	**Bq/Alc/** ***GSTT1***	**<0.0001**	**0.75**	**<0.0001**	**0.75**	**0.10**	**10**
	4^th^ order	Bq/Alc/*GSTM1/GSTT1*	<0.0001	0.78	<0.0001	0.63	0.38	6
ESCC with Methylation index = 0.25–0.50	1^st^ order	Tob	<0.0001	0.70	<0.0001	0.59	0.51	5
	2^nd^ order	Tob/Smk	<0.0001	0.75	<0.0001	0.73	0.05	9
	3^rd^ order	Tob/Smk/*GSTT1*	<0.0001	0.81	<0.0001	0.75	0.05	9
	**4^th^ order**	**Tob/Bq/Smk/** ***GSTT1***	**<0.0001**	**0.83**	**<0.0001**	**0.77**	**0.05**	**9**
ESCC with Methylation index = 0.75–1.0	1^st^ order	Tob	0.0005	0.65	0.0009	0.65	0.26	10
	2^nd^ order	Tob/*GSTT1*	<0.0001	0.71	<0.0001	0.68	0.16	10
	3^rd^ order	Tob/Smk/*GSTT1*	<0.0001	0.74	<0.0001	0.74	0.06	10
	**4^th^ order**	**Tob/Smk/** ***GSTM1/GSTT1***	**<0.0001**	**0.76**	**<0.0001**	**0.75**	**0.06**	**10**

* P-value of 1000 fold permutation test, CVC = Cross Validation Consistency, BQ = Betel Quid, Tob = Tobacco, Smk = smoking, Alc = Alcohol, Interaction models in **Bold** refers to the Best Models selected with maximum CVC, training and testing balance accuracy.

In addition, tobacco chewing was found to be the major 1^st^ order interaction term for both ESCC with methylation index of 0.25–0.50 and 0.75–1.0 in MDR analysis (Table 4). The best predicted model for ESCC with methylation index of 0.25–0.50 comprised of tobacco and betel quid chewing, smoking and null *GSTT1* gene (OR = 25.14 [95% CI = 9.76–64.78]; TBA = 0.77, TrBA = 0.83, CVC = 9/10). For ESCC with methylation index of 0.75–1.0, the best identified model included tobacco chewing, smoking, *GSTM1* and *GSTT1* null genotypes (OR = 11.37 [4.68–27.58], TBA = 0.75, TrBA = 0.76 CVC = 10/10).

### Interaction Entropy Models

**Figure 3:**
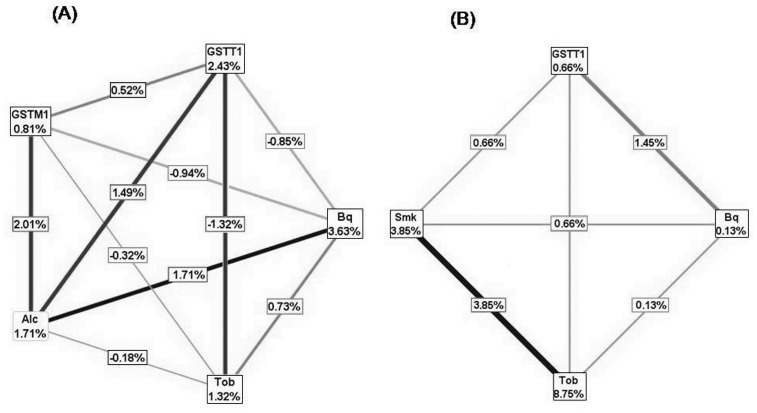
Interaction entropy graphs. (A) ESCC without promoter methylation case-control dataset. (B) ESCC with promoter methylation case-control dataset. All the information gain values (percentages) in the nodes indicate independent main effect of the factors; all the lines (connections) with positive and negative information gain values indicate synergistic interaction and redundancy (lack of interaction) between the factors respectively.

Interaction entropy graphs were constructed on MDR results for ESCC with and without promoter methylation (Figure 3A and 3B). The model constructed for ESCC cases without promoter hypermethylation, and controls had a strong independent effect of betel quid chewing with a synergistic interaction with tobacco chewing (0.73%). Substantial entropy (2.43%) was removed by *GSTT1* null genotype and its interaction with *GSTM1* null genotype (0.52%). Although only a small percentage of entropy in a case-control group was explained by *GSTM1* null genotype (0.81%) and alcohol consumption (1.71%) individually, but their interaction removed 2.02% of the entropy. Moreover, alcohol consumption showed strong synergy with betel quid chewing (1.71%) and *GSTT1* null genotype (1.49%). The model considering ESCC cases with promoter hypermethylation and controls had sizeable entropy removed from the case control group by tobacco chewing (8.48%) and smoking (3.85%) individually, their interaction among themselves (3.85%) as well as with *GSTT1* null genotypes (0.66%); in addition, only a minuscule proportion of the entropy could be explained by betel quid chewing (0.13%) and *GSTT1* null (0.66%) on their own, but a large percentage of the entropy was removed by interaction of these two factors (1.45%).

### False Positive Report Probability (FPRP) of MDR Best Models

The FPRPs of the best models selected in MDR analysis are summarized in Table 5. The best interaction models for ESCC, ESCC with promoter hypermethylation and ESCC with methylation index of 0.25–0.5 were noteworthy even for very low prior probability assumptions (upto10^−3^ to 10^−5^) for detecting ORs of 1.5 and 2.0 for an FPRP value of 0.5. Although, the predicted best models for ESCC without promoter methylation and ESCC with methylation index 0.75–1.0 were noteworthy for low prior probability assumptions (10^−2^ to 10^−3^) when detecting OR = 2.0, but it demonstrated true associations only for high to moderate (0.25–0.10) prior probability assumptions for OR = 1.5.

**Table 5:** False Positive Report Probability for odd ratios of the best models in MDR analysis.

Habits/Gene polymorphism with methylation	Odds Ratio	OR = 1.5 (Prior Probability)						OR = 2.0 (Prior Probability)					
	OR [95% CI] P-value	0.25	0.1	0.01	0.001	0.0001	0.00001	0.25	0.1	0.01	0.001	0.0001	0.00001
ESCCTob/Bq/Smk/*GSTT1*	8.66 [4.41–17.01] P<0.0001	***0.006***	***0.018***	***0.169***	0.672	0.954	0.995	***<10^−4^***	***<10^−4^***	***0.003***	***0.030***	***0.260***	0.778
ESCC with promoterhypermethylationTob/Bq/Smk/*GSTT1*	8.49 [4.23–17.05] P<0.0001	***0.01***	***0.029***	***0.247***	0.768	0.971	0.997	***<10^−4^***	***0.001***	***0.007***	***0.070***	***0.431***	0.883
ESCC withoutpromoterhypermethylationBq/Alc/*GSTT1*	9.88 [3.67–26.54] P<0.0001	***0.152***	***0.351***	0.856	0.984	0.998	1.000	***0.021***	***0.061***	***0.417***	0.878	0.986	0.999
ESCC withmethylation index0.25–0.5Tob/Smk/*GSTT1*	25.14 [9.76–64.78] P<0.0001	***0.027***	***0.076***	***0.447***	0.902	0.989	0.999	***0.001***	***0.003***	***0.02***	***0.234***	0.754	0.968
ESCC withmethylation index0.75–1.0Tob/Smk/*GSTT1*	11.37 [4.68–27.58] P<0.0001	***0.057***	***0.155***	0.668	0.953	0.995	1.000	***0.004***	***0.011***	***0.110***	***0.555***	0.926	0.992

Prior probability range = 0.25–10***^−^*** ^5^ to detect OR = 1.5 or 2.0; α level = observed p-value; ***Bold, italics typing*** = noteworthy association at 0.5 FPRP.

## Discussion

In this case-control study, we examined the association and interaction of various habit related factors and carcinogen metabolizing gene polymorphisms in ESCC and stratified by promoter hypermethylation of multiple tumour suppressor genes using both conventional logistic regression statistics as well as MDR approach. Here, we exploited this non-parametric genetic model free approach to study complex genetic, environmental and epigenetic interactions in ESCC. We identified tobacco consumption as the major risk factor for ESCC and also its probable role in modulating promoter hypermethylation. Moreover, two distinct interaction models for ESCC with and without the promoter hypermethylation advocates discrete gene-gene or gene-environment interactions in both groups.

Tobacco smoking and alcohol consumption are considered among the prominent causes of esophageal cancer worldwide [22], [23]. Although, our study also confirmed tobacco smoking (beedi and cigarette) as a predominant risk factor for ESCC, but highest risk was associated with tobacco chewing in the concerned population. Tobacco is chewed in various forms either alone or with slaked lime or betel quid, and the spit is often swallowed. Like tobacco smoke, smokeless forms of tobacco are also known to contain several carcinogenic compounds, the most potent of which are the tobacco specific *N-*nitrosamines like N’-nitrosonornicotine (NNN), 4-(methylnitrosamino)- 1-(3-pyridyl)-1-butanone (NNK) etc. [24]. Although the environmental and lifestyle factors are undoubtedly associated with ESCC development, but only a minuscule proportion of the exposed individuals actually develops cancer in due course. This is largely due to the differences in inherent carcinogen detoxification capabilities of these individuals, defined by the potency of various carcinogen-metabolizing enzymes that catalyzes the breakdown of the carcinogens present in the body. The *GSTM1* and *GSTT1* genes are responsible for the degradation of several carcinogenic compounds present in tobacco [25]. Null genotypes of *GSTM1* and *GSTT1* were considered to be associated with an increased risk of ESCC [26], [27]. In the present study; null genotypes of both *GSTM1* and *GSTT1* genes were higher in cases than controls, imparting 1.44 folds and 1.74 folds risk towards developing ESCC respectively. A study from Chinese population documented 2.17 folds increased risk of ESCC in *GSTM1* null individuals than *GSTM1 carriers* [27]. However, a pooled analysis of 11 studies could ascertain only a modest increase in risk of ESCC in *GSTM1* null genotype carriers [OR = 1.197 (95% CI = 0.846–1.692)] [6], and two others failed to establish any association between *GSTM1* and *GSTT1* polymorphisms and risk of ESCC [28], [29].

The best model for ESCC in MDR analysis was the interaction of tobacco chewing, betel quid chewing, smoking and *GSTT1* null genotype with an OR of 8.66 [95% CI = 4.41–17.01, p<0.0001]. Although no prior report used MDR for studying these risk factors in ESCC, but an earlier case-control study using logistic regression conducted on the population of north-east India have documented the highest risk of esophageal cancer in betel quid and tobacco chewers with smoking habit (OR = 15.3 in males, OR = 27.4 in females) [17]. A study conducted on a South Asian population established a 21.4 fold increased risk of ESCC in betel quid and tobacco chewers who smoked cigarettes [30]. However, both the studies did not take genetic factors into consideration.

The role of promoter hypermethylation of tumour suppressor genes is recognized as one of the key events in instigation and progression of cancer by repressing the expression of the corresponding genes. Here, we studied promoter methylation status of key tumour suppressor genes involved in different cellular pathways and thought to be important in cancer development and progression, namely, *p16* (cell cycle regulation), *DAPK* (apoptosis) *BRCA1* (DNA repair) and *GSTP1*(protection of DNA). Although, promoter methylation of *p16, DAPK, GSTP1* and *BRCA1* genes are frequent events in several carcinomas, including ESCC [26], [31]–[34], but very few studies considered these genes together in ESCC. In our study group, 37.5%, 61.60%, 58.92% and 20.53% of the ESCC tumours had *p16, DAPK, GSTP1* and *BRCA1* promoter methylation respectively, which was significantly higher than adjacent normal tissues. A study conducted by Guo et al. [35] found a comparatively higher proportion of *p16* (52%) and a lower percentage of *DAPK* (24%) promoter hypermethylation in ESCC tumours, which might be due to ethnic variations; however, they reported a similar proportion of *BRCA1* promoter methylation (28%).

Based on the promoter hypermethylation status, the cases were further categorized as ESCC with and without promoter hypermethylation. Tobacco chewing and smoking were the main risk factors for ESCC with promoter hypermethylation of all the four genes under study when compared to controls. Further classifying the cases according to methylation index, tobacco chewing had the highest risk of ESCC having methylation index 0.25–0.50, followed by betel quid chewing. However, cases with higher methylation index (0.75–1.0) had strongest associations with tobacco consumption, both chewing and smoking, with an odds ratio of 6.04 and 5.29 respectively. The same was reflected in MDR, as tobacco chewing was the best one factor model in ESCC with promoter hypermethylation overall and also stratified by methylation index. A similar study from Indian oral cancer patients found a significantly higher percentage of *p16* and *DAPK* promoter methylation in tobacco chewers as compared to non-chewers [35]. The fact that certain tobacco specific nitrosamines and poly-aromatic hydrocarbons (PAHs) like NNK, Benzo[*a*]pyrene etc. are capable of modulating DNA methylation is evident to both in-vitro as well as human studies. In previous studies, NNK was found to induce hypermethylation of multiple tumour suppressor genes like *p16*, *DAPK*, *Rarβ* etc. in liver and lung tumours of rat and mouse models [11], [36], [37]. A recent study combining cell, animal and clinical lung cancer tissues as a modelfound that, NNK attenuates DNMT1 degradation and also induces its nuclear accumulation resulting in subsequent hypermethylation of promoters of tumour suppressor genes [10]. Benzo[a]pyrene present in tobacco is converted to its carcinogenic form BPDE (benzo[a]pyrenediolepoxide) by the phase I enzymes CYP1A1, CYP1B1, etc., which are further metabolized by the GSTs. In a study on esophageal cancer cells, BPDE was found to suppress *Rarβ* expression via promoter hypermethylation by recruiting DNMT3A [38]. Moreover, DNA repair and carcinogen metabolising gene polymorphisms are believed to predispose cells towards promoter hypermethylation of genes [15]. In this study, significant association was observed between *GSTM1* null genotypes and promoter methylation of *p16, DAPK* and *GSTP1* genes, whereas, null genotypes of *GSTT1* gene had the association *p16* and *BRCA1* methylation only. In addition, both *GSTM1* and *GSTT1* null polymorphisms were significantly associated with ESCC having MI = 0.75–1.0. In a case-control study on lung cancer, null genotype of *GSTM1* has been found to increase the risk of promoter hypermethylation of *DAPK* and *Rarβ*. Moreover, they also identified significant interaction of tobacco smoking and null *GSTM1* genotype in modulating promoter hypermethylation of multiple TSGs [12]. In our study, the interaction of tobacco and betel quid chewing, smoking and null *GSTT1* genotype was the best model for both ESCC with promoter hypermethylation and MI = 0.25–0.50 in MDR. Additionally, the interaction of tobacco chewing, smoking, *GSTT1* and *GSTM1* null genotypes was the optimal model for ESCC with MI = 0.75–1.0. This further supports the hypothesis that a complex interaction is likely to interplay between tobacco-related habits and carcinogen metabolizing gene polymorphisms towards promoting aberrant DNA methylation in ESCC.

In ESCC without promoter hypermethylation, betel quid chewing was the most prominent risk factor, followed by alcohol drinking and *GSTT1* polymorphism. The same was reflected in MDR, as betel quid chewing was the best one factor model and the interaction of betel quid, alcohol and *GSTT1* null genotype was the finest model recognized. The alcohol-betel quid interaction was not only found to modify the risk of ESCC in earlier studies, but was also associated with methylation of certain genes [23], [39]. However, we were not able to establish any strong association of betel quid chewing or alcohol consumption with promoter hypermethylation, except for a moderate association of betel quid chewing with ESCC having a comparatively lower methylation index (MI = 0.25–0.50).

Entropy graphs were drawn for visualization and interpretation of MDR interactions. Tobacco chewing and smoking showed highest individual effects as well as strongest synergistic effects among each other in ESCC with promoter hypermethylation, supporting the role of tobacco carcinogens in promoting DNA methylation in ESCC. In ESCC without promoter hypermethylation, interactions of alcohol consumption with betel quid chewing and *GSTT1* null genotype was most striking.

There are both strengths as well as limitations to this study. This is the first case-control study on the association and interaction of environmental, genetic and epigenetic factors in ESCC using both LR as well as MDR approaches. We further strengthened the data by testing the robustness and consistency of the best interaction model obtained from MDR using false positive report probability (FPRP) analysis. The best models for total data set of ESCC and ESCC with promoter hypermethylation showed excellent reliability even at low prior probabilities. The relatively small sample size in our study might be a drawback for predicting high-order interactions; however, MDR is known to reliably predict interactions in spite of for low sample sizes. Moreover, while studying the habit-related factors, the duration or frequency of use was not considered, as such the dose-related response could not be established.

In conclusion, our study not only confirmed tobacco consumption as the main risk factor of ESCC in NE India, but also indicated its possible interaction with carcinogen metabolizing genes towards modulating promoter hypermethylations of TSGs. Nevertheless, it is only a pilot association study and requires in-depth investigations involving larger populations and in-vitro models to establish the role of these interactions in ESCC.

## Supporting Information

Figure S1 **Comparison of promoter methylation profile of** ***p16, DAPK, GSTP1*** **and** ***BRCA1*** **genes of 30 ESCC tissues with their corresponding normal tissues.** Each column represents a gene indicated on top. Each row indicates individual patients. The number indicated in the fifth column corresponds to the patient ID. Black rectangles are methylated samples; white rectangles are unmethylated samples.
(JPG)
