# Supplementary figures and images for: Epigenetic, Genetic and Environmental Interactions in Esophageal Squamous Cell Carcinoma from Northeast India
Source: PLoS One. 2013 Apr 15;8(4):e60996. doi: 10.1371/journal.pone.0060996 (PMC3626640; doi:10.1371/journal.pone.0060996)

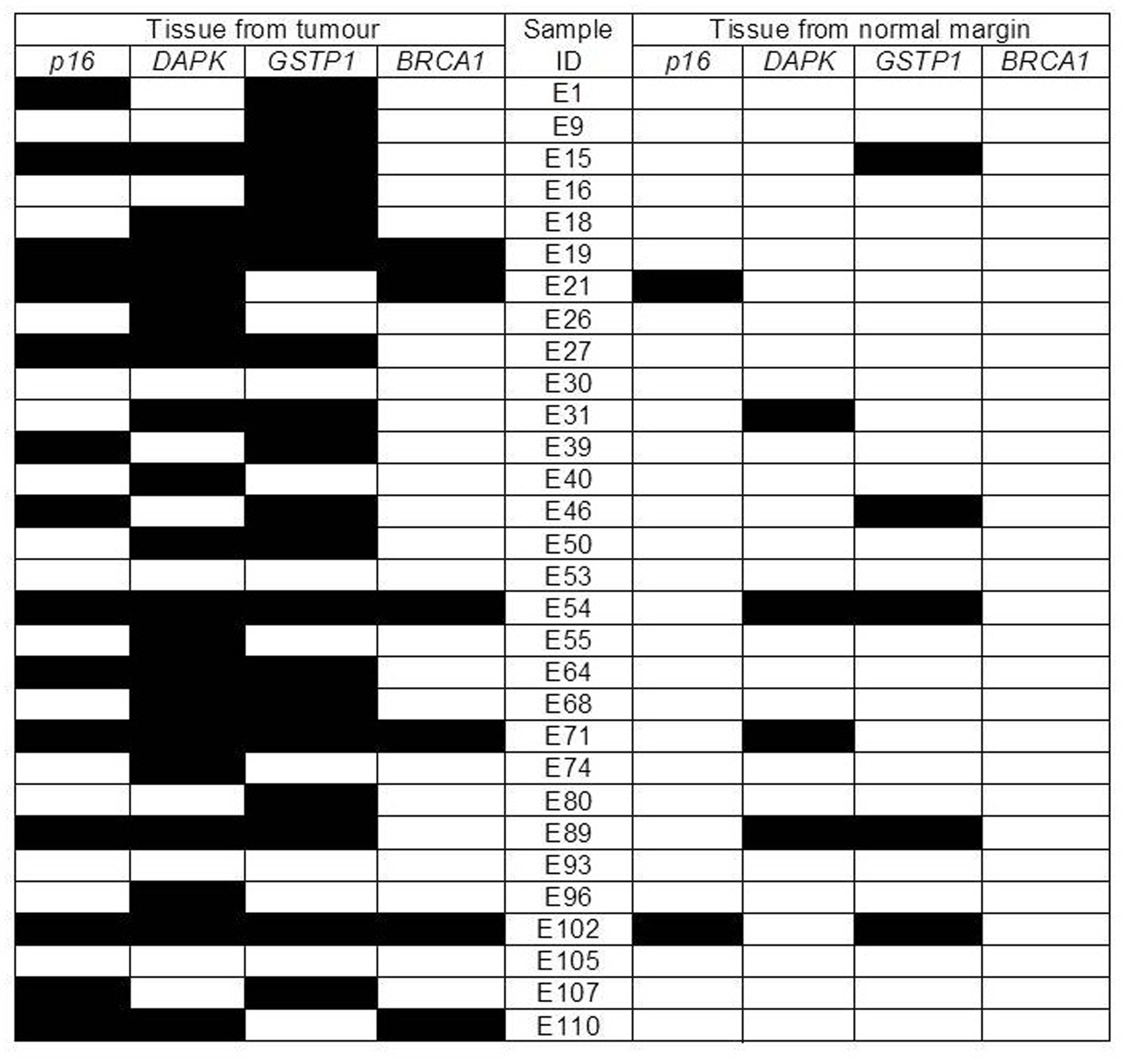

Supplement: Figure S1 — Comparison of promoter methylation profile of p16, DAPK, GSTP1 and BRCA1 genes of 30 ESCC tissues with their corresponding normal tissues. Each column represents a gene indicated on top. Each row indicates individual patients. The number indicated in the fifth column corresponds to the patient ID. Black rectangles are methylated samples; white rectangles are unmethylated samples. (JPG) [file pone.0060996.s001.jpg]
